# Investigation of Gas-Sensing Property of Acid-Deposited Polyaniline Thin-Film Sensors for Detecting H_2_S and SO_2_

**DOI:** 10.3390/s16111889

**Published:** 2016-11-10

**Authors:** Xingchen Dong, Xiaoxing Zhang, Xiaoqing Wu, Hao Cui, Dachang Chen

**Affiliations:** 1 State Key Laboratory of Power Transmission Equipment & System Security and New Technology, Chongqing University, Chongqing 400044, China; 20141113009t@cqu.edu.cn (X.D.); cqu_cuihao@cqu.edu.cn (H.C.); 2 School of Electrical Engineering, Wuhan University, Wuhan 430072, China; 2016202070027@whu.edu.cn; 3 Fuyang Power Supply Company, State Grid Anhui Electric Power Company, Fuyang 236000, China; xiaoqingwu08@gmail.com

**Keywords:** gas-insulated switchgear, partial discharge, polyaniline, acid deposition, gas-sensing property

## Abstract

Latent insulation defects introduced in manufacturing process of gas-insulated switchgears can lead to partial discharge during long-time operation, even to insulation fault if partial discharge develops further. Monitoring of decomposed components of SF_6_, insulating medium of gas-insulated switchgear, is a feasible method of early-warning to avoid the occurrence of sudden fault. Polyaniline thin-film with protonic acid deposited possesses wide application prospects in the gas-sensing field. Polyaniline thin-film sensors with only sulfosalicylic acid deposited and with both hydrochloric acid and sulfosalicylic acid deposited were prepared by chemical oxidative polymerization method. Gas-sensing experiment was carried out to test properties of new sensors when exposed to H_2_S and SO_2_, two decomposed products of SF_6_ under discharge. The gas-sensing properties of these two sensors were compared with that of a hydrochloric acid deposited sensor. Results show that the hydrochloric acid and sulfosalicylic acid deposited polyaniline thin-film sensor shows the most outstanding sensitivity and selectivity to H_2_S and SO_2_ when concentration of gases range from 10 to 100 μL/L, with sensitivity changing linearly with concentration of gases. The sensor also possesses excellent long-time and thermal stability. This research lays the foundation for preparing practical gas-sensing devices to detect H_2_S and SO_2_ in gas-insulated switchgears at room temperature.

## 1. Introduction

Latent insulation defects inevitably appearing in gas-insulated switchgear (GIS) will cause partial discharge (PD) in GIS equipment when the equipment has been running for a long time. More seriously, such defects might result in insulation failure of GIS equipment. Under the energy of partial discharge, SF_6_ gas inside GIS will decompose into products like H_2_S, SO_2_, etc. [1,2,3,4]. Detecting SF_6_ decomposition products by gas sensors has broad application prospects in on-line monitoring and fault diagnosis field of electric equipment [5,6]. Through the method of detecting H_2_S, SO_2_, and their concentrations using installed gas sensors, the operating status and insulation level of GIS equipment can be evaluated, thus avoiding the sudden fault caused by latent insulation defects [7,8].

So far, gas sensors which possess good selective and stable properties have been attracting much more attention for various applications [9,10,11]. Gas sensors under study for on-line monitoring and fault diagnosis of electrical equipment mainly include carbon nanotube gas sensors [12,13], titanium nanotube gas sensors [14,15], as well as graphene gas sensors [16,17]. Some research results related to GIS partial discharge monitoring field have been achieved [18].

Polyaniline (PANI) gas sensing material has drawn researchers’ attention in the field of sensor application due to its unique sedimentary mechanism, structure diversification, cheap raw materials, and simple synthetic process. A British scholar, N.E. Agbor, synthetized PANI gas sensitive membrane in neutral environments and successfully detected NO_x_, CO, CH_4_, etc. [19]. H. Liu built single polyaniline nanowire on gold electrode using deposition technique and gained rapid and reversible changes in resistance when detecting NH_3_ at the concentration of 0.5 μL/L [20]. Shabnam Virji studied the effect of camphor sulfonic acid deposition on gas-sensitive property of PANI to H_2_, with the influence of humidity and oxygen explored as well [21]. J. Bhadra prepared polystyrene-deposited PANI of different deposition quantity and investigated gas-sensing characteristics for detecting CO_2_ gas [22]. Based on what has been mentioned above, it is necessary to carry out research on PANI gas sensors in the field of GIS partial discharge monitoring.

According to research findings, deposition of proton acid in PANI materials can increase the number of carries inside these materials, thus significantly improving the conductivity of polymer. The size of inorganic acid molecule is small and the molecule is prone to spread in the polyaniline chain, which means the deposition process is simple, but the deposited products have poor solubility and stability; macromolecular organic acid can improve the solubility and stability of products but electrical conductivity of polymer is less than that of inorganic acid deposited polymer. In the previous work, gas sensing properties of PANI with hydrochloric acid deposited have been studied. In order to investigate influences of different acids on gas-sensing properties of PANI, both inorganic acid of small molecule size, hydrochloric acid (HCl), and macromolecular organic acid, sulfosalicylic acid (SSA), were used as sedimentary elements. PANI polymers with HCl deposition only and with HCl plus SSA deposition were prepared using chemical oxidative polymerization method, namely PANI-SSA and PANI-HCl/SSA, respectively. Gas-sensing performances of these two types of sensors when exposed to H_2_S and SO_2_, two important SF_6_ decomposed components, were studied at room temperature. Performances of these two newly-prepared sensors were compared with that of PANI-HCl gas sensors which had been studied in the previous work [23].

## 2. Experimental Section

### 2.1. Preparation of PANI-SSA and PANI-HCl/SSA

**Figure 1:**
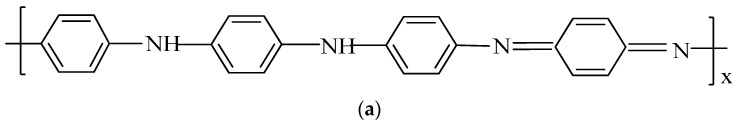

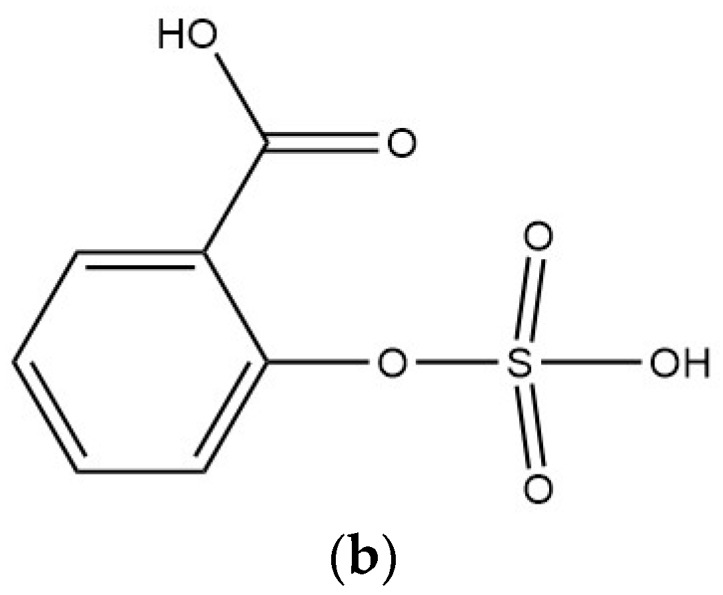
Schematic diagram of PANI and SSA molecules. (**a**) Molecular structure of polyaniline; (**b**) Molecular structure of sulfosalicylic acid.

Figure 1 shows the structures of polyaniline and sulfosalicylic acid molecule. In the process of proton acid deposited on polyaniline, the number of electrons on the main chain of polyaniline frame does not change, with protons merely entering the main chain and making the chain positively charged. In order to maintain neutral, the anions of protonic acid also enter the main polymer chain. The protons which go into the imino group, thus forming polaron and dipole, delocalize to the PI bond of PANI. This process will increase the number of carriers in the molecular chain and enhance the migration rate of carriers. The conductivity of PANI after deposition can increase 9~10 orders of magnitude.

The experimental process of synthetizing PANI-HCl/SSA and PANI-SSA using the method of chemical oxidative polymerization is as follows: since aniline tends to be oxidized if stored in the air for long time, quadratic distillation purification must be carried out in the presence of zinc powder before use. First of all, 1 mol/L composite acid of which the molar ratio of hydrochloric acid to sulfosalicylic acid was 4:1, that is, *n* (HCl):*n* (SSA) = 4:1, was prepared. Then dissolved 0.1 mol aniline monomers into 50 mL composite acid of 1 mol/L and stirred with a glass rod to make sure that it was dispersed adequately. The following process was to make 0.1 mol ammonium persulfate dissolve in 1 mol/L composite acid, with molar ratio of aniline to ammonium persulfate equaling 1:1. Put the mixed acid solution which contained aniline monomers into magnetic stirring apparatus and slowly drop ammonium persulfate solution into it while stirring. The duration of dropping ammonium persulfate was controlled to about 30 min, and the polymerization reaction temperature was controlled at 0~5 °C, with reaction time being 8 h.

After vacuum filtration and product collection, the filter cake is washed repeatedly with distilled water and anhydrous ethanol respectively until the filtrate is colorless. Dry it in a vacuum oven at 60 °C for 24 h and grind it into powder form so that hydrochloric acid and sulfosalicylic acid deposited PANI can be obtained, named PANI-HCl/SSA. PANI with only sulfosalicylic acid deposited is denoted as PANI-SSA.

### 2.2. Characterization of PANI-SSA and PANI-HCl/SSA

**Figure 2:**
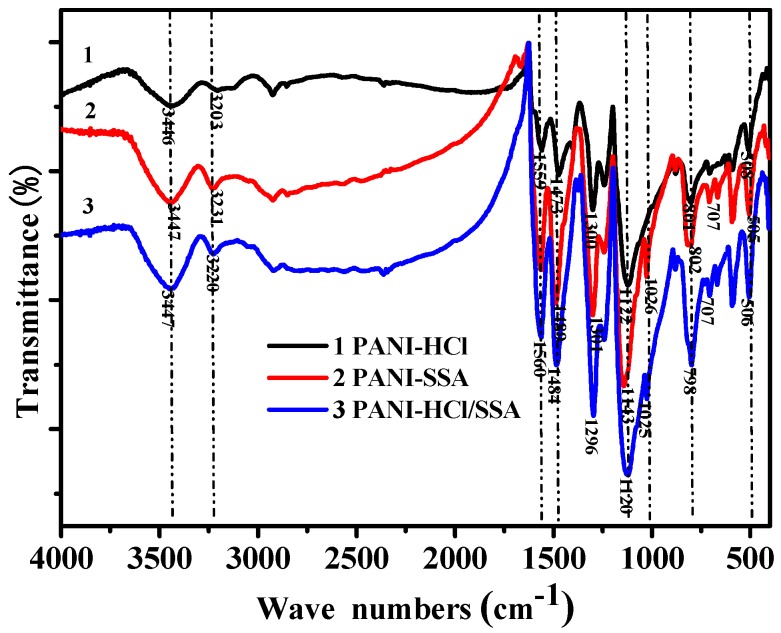
FTIR spectrum of different acid-deposited PANI.

Fourier transform infrared spectroscopy (FTIR) of different acid deposited PANI is shown in Figure 2. It can be seen that PANI-SSA and PANI-HCl/SSA have a new absorption peak at around 1025 cm^−1^ compared with PANI-HCl. This absorption peak is probably the stretching vibration characteristic peak of O=S=O in -SO_3_H, which indicates that sulfonate group exists in PANI after acid deposition. Details of infrared characteristic peaks of PANI with different acid deposition are listed in Table 1.

**Figure 3:**
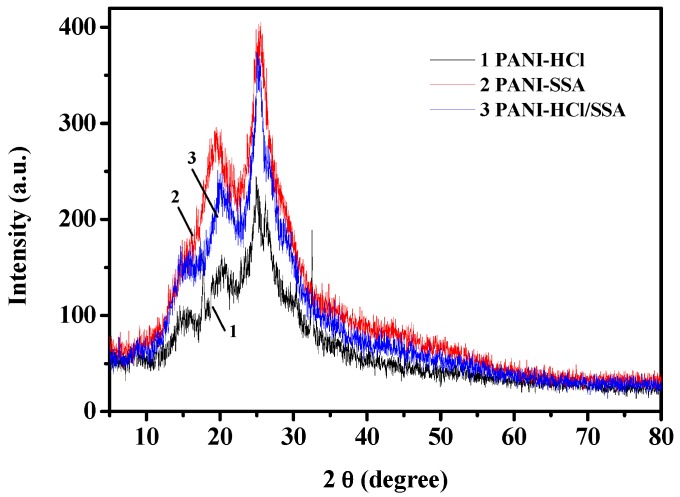
XRD pattern of different types of acid-deposited PANI.

The X-ray diffraction (XRD) of PANI with sulfosalicylic acid and mixed acid deposition is shown in Figure 3, with XRD of hydrochloric acid deposited PANI added as well. Through analyzing the XRD of the sample, two samples of sedimentary state show a certain degree of crystallinity. Different types of acid deposition make the strength and width of the diffraction peak differ from each other. Research shows that the narrower the width of diffraction peak and the greater the intensity, the better the crystallinity; the relative height of diffraction peak can indirectly reflect crystal orientation degree, which means that the smaller the relative height, the better the degree of crystal orientation. The intensity of diffraction peak can also indirectly reflect ordering degree of crystal. The higher the intensity is, the greater degree of ordering in the crystal. The electrical conductivity of the sample is directly affected by the crystallinity and crystal degree of ordering.

As shown in Figure 3, the samples have similar diffraction peaks. To be more precise, there are sharp diffraction peaks appearing between 2θ = 20° and 2θ = 25°, which indicates that the materials are fractionally crystalline. However, the details of diffraction peaks of polyaniline with different acid deposition are different from each other, especially the details of peak intensity. The XRD of PANI-HCl/SSA shows that intensity of diffraction peak is relatively big, with the narrowest width. This indicates that the crystallinity and crystal degree of ordering are better.

**Figure 4:**
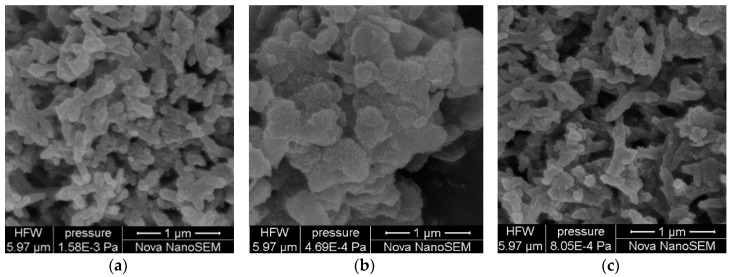
SEM image of PANI-HCl, PANI-SSA, and PANI-HCl/SSA. (**a**) PANI-HCl; (**b**) PANI-SSA; (**c**) PANI-HCl/SSA.

Figure 4 compares the scanning electron microscope (SEM) images of PANI-SSA, PANI-HCl/SSA, as well as PANI-HCl. Because the anion of organic sulfonic acids is bigger than that of hydrochloric acid, when it enters into the main chain of PANI, the structure of the chain is more stretched and larger chain spacing is formed. As a result, the chain-to-chain interaction of polymer is less effective. By comparing the SEM images in Figure 4, it can be inferred that, under the same condition of polymerization deposition, it is easy to obtain short-rod structure through small molecule inorganic acid deposition, and plate-shape structure through organic acid deposition. Under the synergy of both HCl and SSA deposition, the product of compound acid deposition shows a rod-like structure.

### 2.3. Schematic Diagram of PANI-SSA and PANI-HCl/SSA Sensors

**Figure 5:**
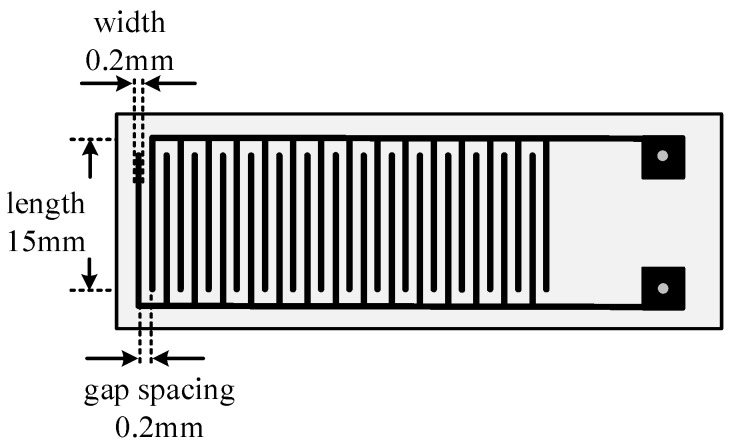
Schematic diagram of a PANI gas sensor.

An interdigital electrode sensor with length of 5 cm, width of 1.5 cm, and effective area of 4.8 cm^2^ was chosen in this paper. The width of electrode and gap spacing of adjacent electrodes were both 0.2 mm. The schematic diagram is shown in Figure 5. The first step is to dissolve HCl/SSA-deposited PANI powder into anhydrous ethanol and conduct ultrasonic oscillation process to disperse the above power adequately. The concentration of the solution is 1 mol/L. Then carry out homogeneous dispersion by making solution spread on the surface of interdigital electrodes. The next step is to dry the sensor in a drying oven at constant temperature of 80 °C. Finally repeat coating and drying for 3~5 times until obtaining deposited PANI thin film sensors with uniform surface.

## 3. Results and Discussion

### 3.1. Gas-Sensing Property Test of PANI-SSA

#### 3.1.1. Gas-Sensing Response of PANI-SSA to H_2_S

The sensors utilized in this paper are interdigital-electrode and resistance-type sensors. The resistance change (*S*) is used to characterize the sensitivity of sensors to target gases, which can be defined as:

$$S=\frac{R-R_{0}}{R_{0}}\times 100\%=\frac{\Delta R}{R_{0}}\times 100\%$$

**Figure 6:**
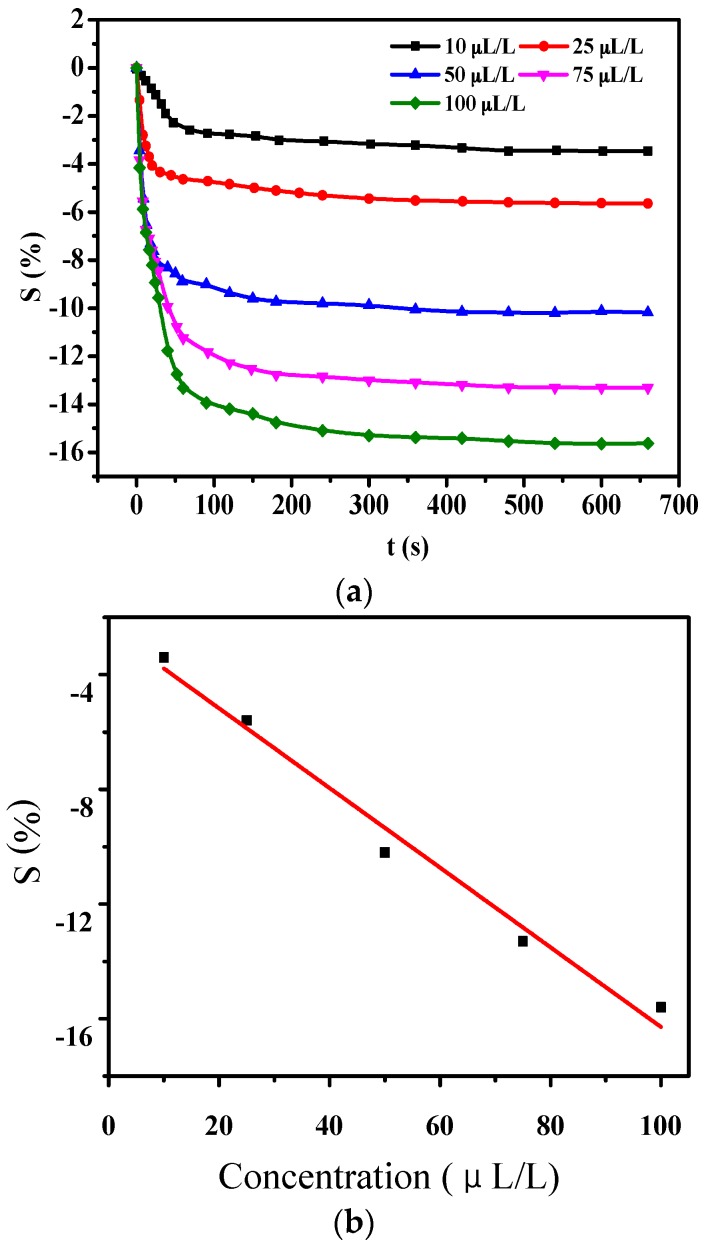
Gas-sensing response of PANI-SSA sensor to H_2_S of different concentration. (**a**) Response to a range of concentration of H_2_S; (**b**) Liner relationship between sensitivity and gas concentration.

As shown in Figure 6a, it can be found that the figures for sensitivity of PANI-SSA are −3.4%, −5.6%, −10.1%, −13.2%, and −15.6% when concentrations of H_2_S were controlled at 10 μL/L, 25 μL/L, 50 μL/L, 75 μL/L, and 100 μL/L, respectively. The stable time of gas-sensing response is about 300 s. The liner fitting dependence between sensitivity of sensors and concentration of H_2_S could be described as *y* = −0.11537*x* − 1.96098 with the linearly dependent coefficient *R*^2^ equaling 0.98672.

#### 3.1.2. Gas-Sensing Response of PANI-SSA to SO_2_

**Figure 7:**
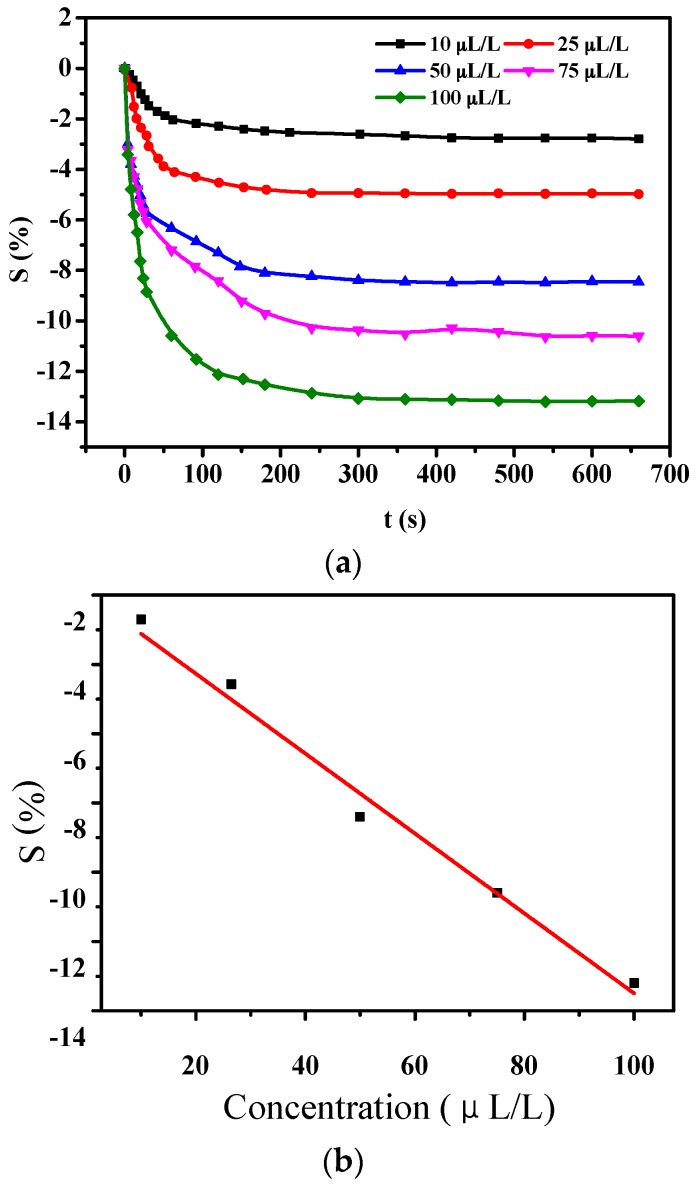
Gas-sensing response of PANI-SSA sensor to SO_2_ of different concentration. (**a**) Response to various concentration of SO_2_; (**b**) Liner dependence between sensitivity and gas concentration.

Figure 7 shows gas-sensing response curves of sensors to SO_2_ of different concentrations. It could be obtained from Figure 7a that the sensitivity to SO_2_ of 10 μL/L, 25 μL/L, 50 μL/L, 75 μL/L, and 100 μL/L are −2.7%, −4.6%, −8.4%, −10.6%, and −13.2%, respectively. The stable time of gas-sensing response is about 300 s. The liner fitting dependence shown in Figure 7b could be described as *y* = −0.13889*x* − 2.39756 with *R*^2^ = 0.97875, which shows relatively good linearity.

#### 3.1.3. Stability Test of PANI-SSA Sensors

**Figure 8:**
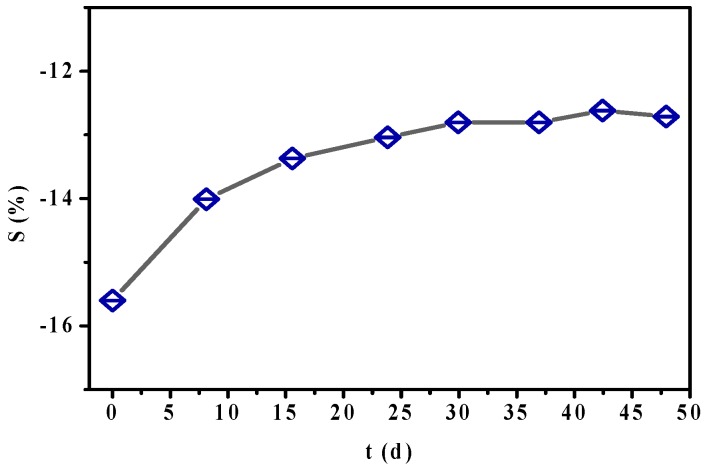
Stability curves of PANI-SSA sensor to H_2_S.

The whole working life of sensors is a significant parameter that can be used to measure their performance. The gas responses of sensors were continuously tested exposed to 100 μL/L H_2_S for 49 days with every 6 days as a test period. The stability was defined as a ratio between actual sensitivity and initial sensitivity. The results were showed in Figure 8, from which it could be concluded that the absolute value of resistance change rate decreased from 15.6% to 12.7% and stability reached 81.4% through calculation.

**Figure 9:**
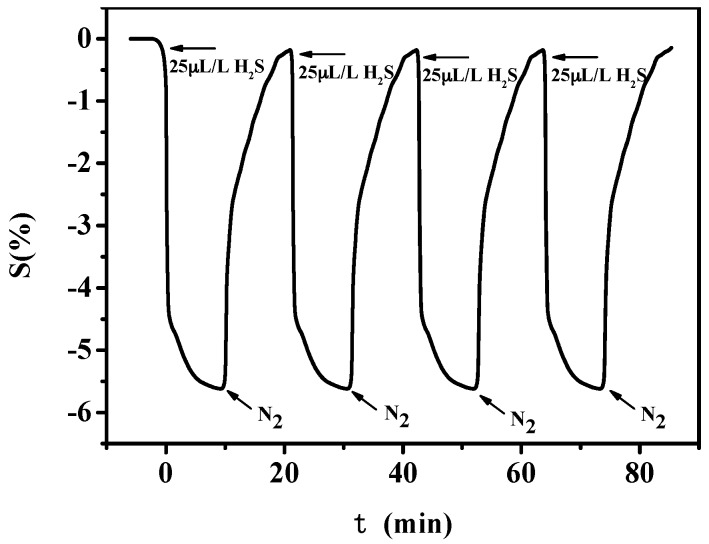
Reversibility curve of PANI-SSA sensors to H_2_S.

Figure 9 shows the gas-sensing response of PANI-SSA to 25 μL/L H_2_S under multiple exposure cycles. It can be seen that the largest sensitivity keeps a stable value and the gas-sensing response returns to the initial value after exposure to N_2_, which means that the prepared sensor can recover from the exposure of targeted gases.

### 3.2. Gas-Sensing Property Test of PANI-HCl/SSA

#### 3.2.1. Gas-Sensing Response of PANI-HCl/SSA to H_2_S

**Figure 10:**
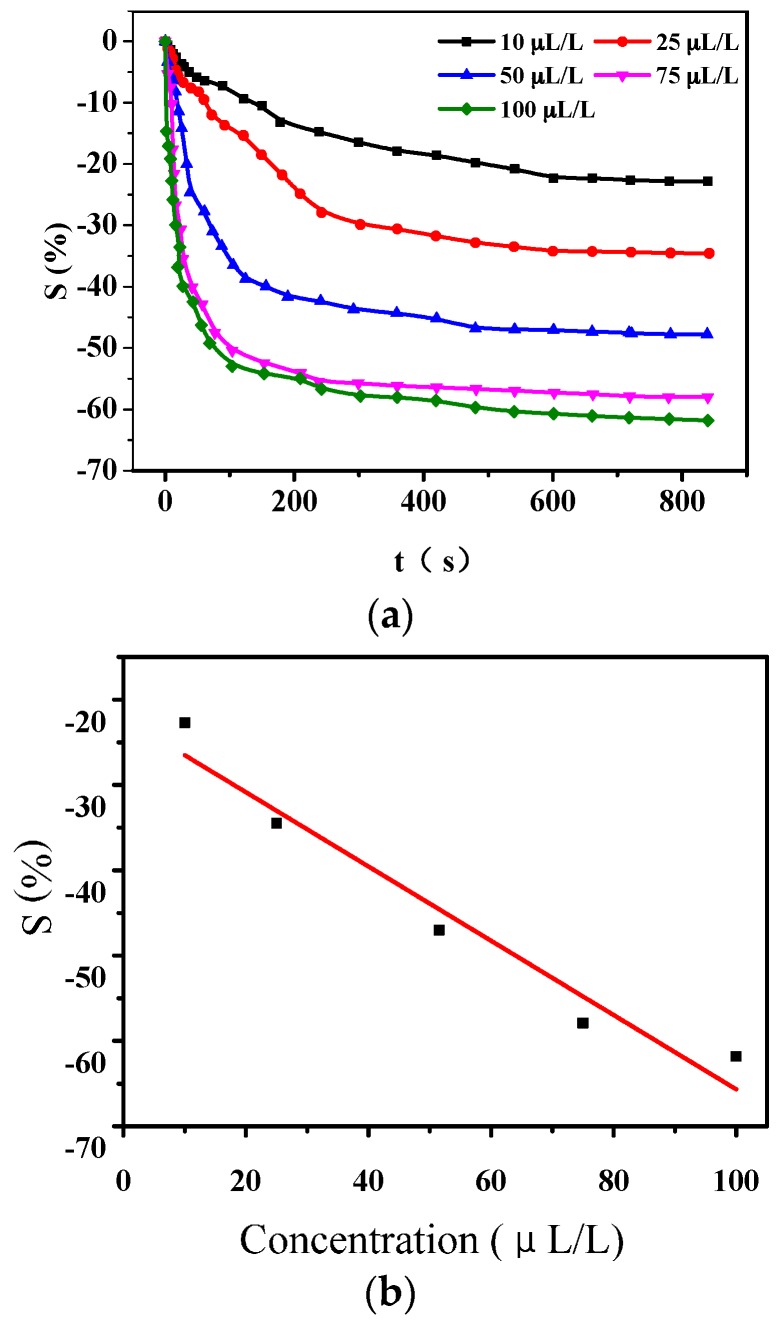
Gas-sensing response of PANI-HCl/SSA sensor to H_2_S of different concentration. (**a**) Response to various concentration of H_2_S; (**b**) Liner dependence between sensitivity and gas concentration.

Based on the definition of gas-sensing response of sensors, when applied to detect H_2_S of various concentration of 10 μL/L, 25 μL/L, 50 μL/L, 75 μL/L, and 100 μL/L, the sensitivities of compound acid deposited PANI sensors were −22.7%, −34.5%, −47.6%, −57.9%, and −61.8%, respectively, shown in Figure 10a. The stable time of gas-sensing response is about 350 s. The liner dependence shown in Figure 6b could be described as *y =* −0.43512*x* − 22.15366, with *R*^2^ = 0.93608.

#### 3.2.2. Gas-Sensing Response of PANI-HCl/SSA to SO_2_

**Figure 11:**
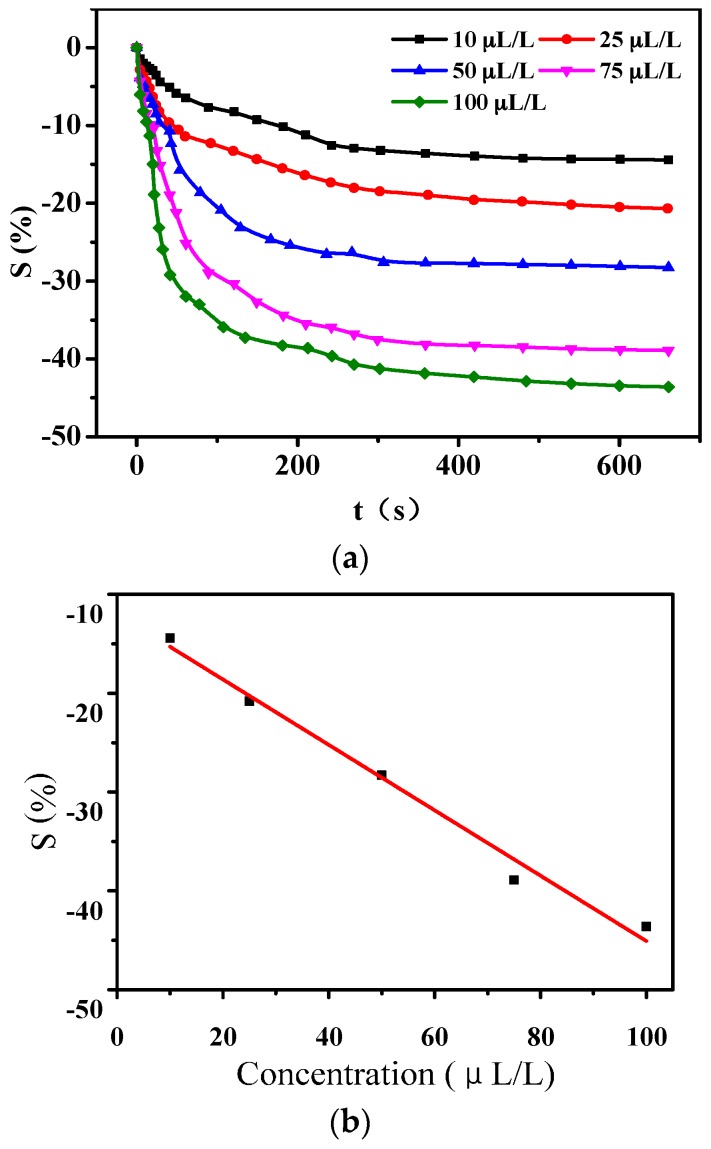
Gas-sensing response of PANI-HCl/SSA sensor to SO_2_ of different concentration. (**a**) Response to various concentration of SO_2_; (**b**) Liner dependence between sensitivity and gas concentration.

As shown in Figure 11, the sensitivities of PANI/SSA to different concentration of SO_2_ are −14.4%, −20.8%, −28.3%, −38.9%, and −43.6%, respectively. The stable time of gas-sensing response is about 500 s. The liner relationship could be described as *y* = −0.33105*x* − 11.98537, with *R*^2^ = 0.98261.

The sensitivity of PANI-HCl/SSA to H_2_S and SO_2_ of 100 μL/L is −61.8% and −43.6%, respectively. Additionally, H_2_S is a type of characteristic gas that only appears under serious discharge fault and overheating condition, while SO_2_ is a main decomposed product when normal partial discharge occurs inside electrical equipment. That is to say, these two gases are generated in different stages with different production rates. So it could be concluded that cross interference does not happen easily in the detection of these two gases.

#### 3.2.3. Stability Test of PANI-HCl/SSA Sensors

**Figure 12:**
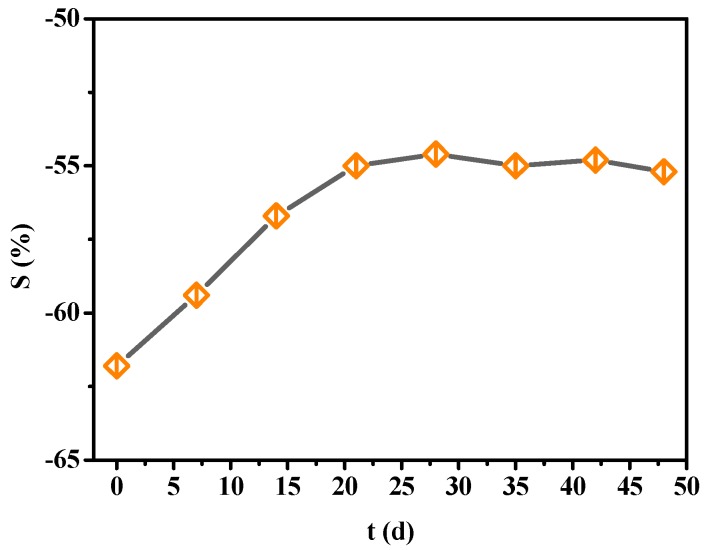
Stability curves of PANI-HCl/SSA sensor to H_2_S.

Similarly, gas responses of PANI-HCl/SSA sensors to 100 μL/L H_2_S were continuously tested for 49 days, at intervals of every 6 days. The results shown in Figure 12 present that the absolute value of resistance change rate decreases from 61.8% to 55.2% and the stability reaches 89.3%.

**Figure 13:**
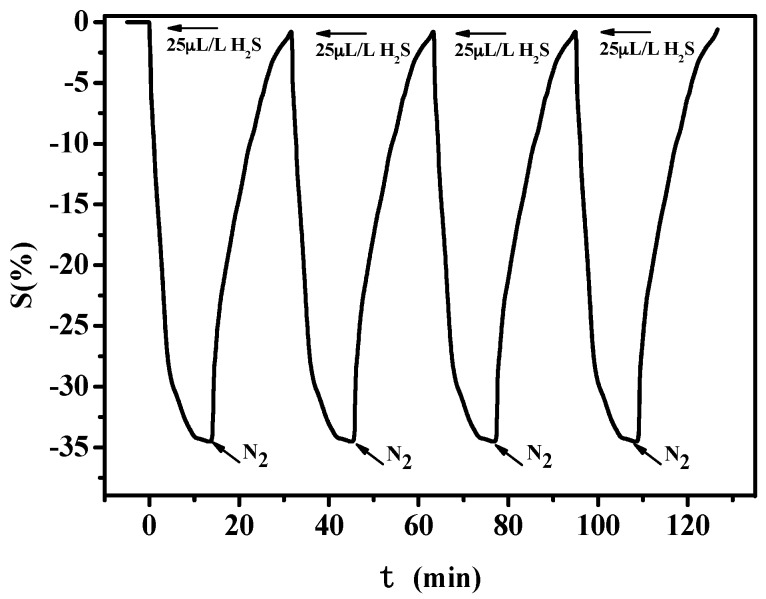
Reversibility curve of PANI-HCl/SSA sensors to H_2_S.

Figure 13 shows the gas-sensing response of PANI-HCl/SSA to 25 μL/L H_2_S under multiple exposure cycles. Similar conclusions can be obtained that PANI-HCl/SSA possesses good recovery characteristics with PANI-SSA.

### 3.3. Gas-Sensing Property Comparison of PANI-SSA, PANI-HCl/SSA, and PANI-HCl

#### 3.3.1. Comparison of Gas-Sensing Response Characteristics and Mechanism Analysis

According to the liner relationship curve between sensitivity and gas concentration, gas-sensing response of prepared PANI gas sensors are listed in Table 2. The recommended values from CIGRE are ≤1.0 μL/L for SO_2_, and ≤0.5 μL/L for H_2_S.

Depositions of different proton acids have distinct influences on the gas-sensing characteristics of polyaniline. Therefore, the influence on both response time and sensitivity of sensors were analyzed as follows.

**Figure 14:**
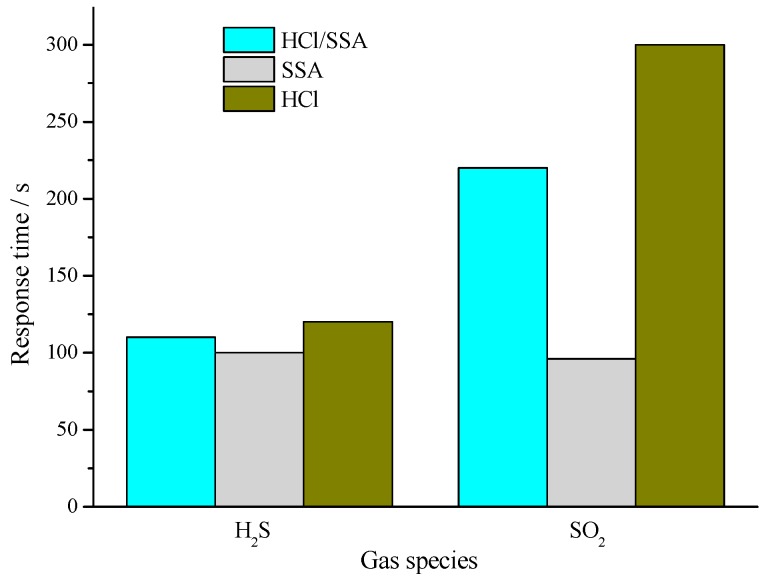
Response time of PANI-HCl and PANI-HCl/SSA sensor to H_2_S and SO_2_ of 100 μL/L.

**Figure 15:**
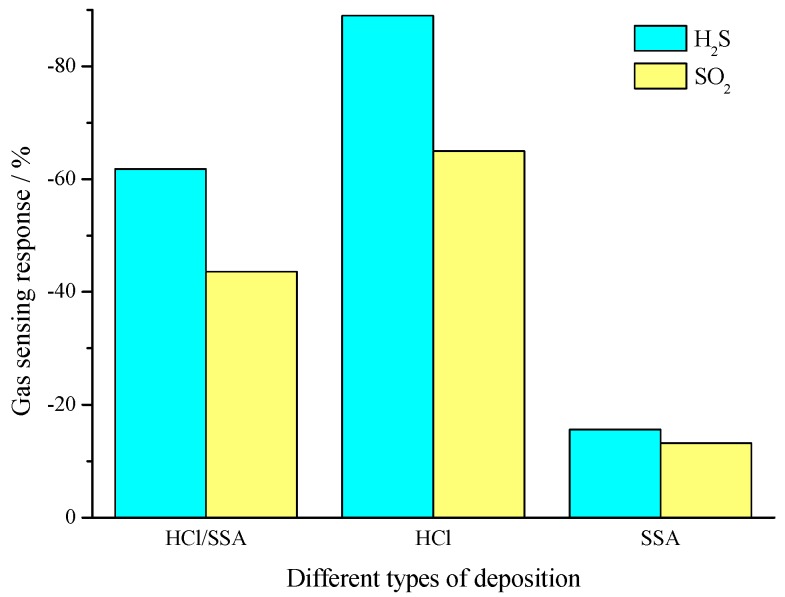
Gas-sensing sensitivity of PANI-HCl and PANI-HCl/SSA sensors to H_2_S and SO_2_ of 100 μL/L.

Figure 14 shows the response time of PANI-HCl, PANI-SSA, and PANI-SSA sensors to H_2_S and SO_2_ of 100 μL/L. It could be found that PANI-SSA sensors possess the least response time, and the response time of PANI-HCl/SSA sensors is slightly longer, with PANI-HCl sensors showing the longest response time. Figure 15 presents the sensitivity of three types of sensors, PANI-HCl, PANI-SSA, and PANI-SSA sensors, to 100 μL/L H_2_S and SO_2_. It could be observed that PANI-HCl has the best sensitivity to H_2_S and SO_2_, and PANI-HCl/SSA takes second place while PANI-SSA is the last.

**Figure 16:**
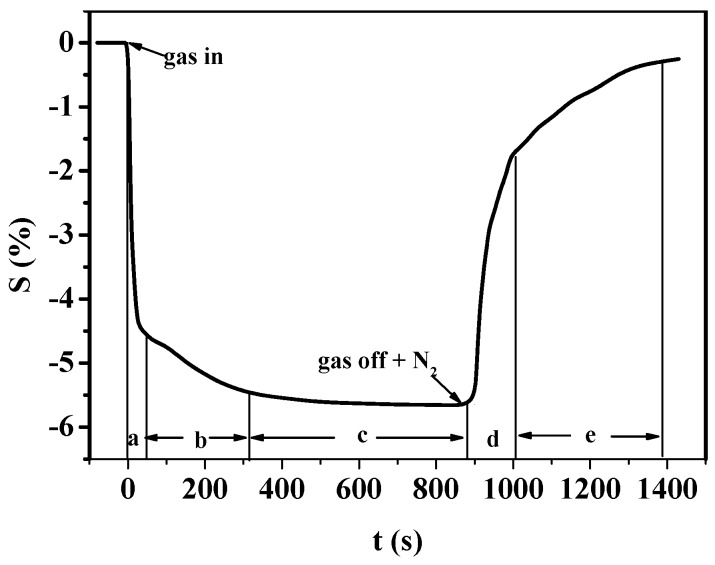
Response and recovery curve of PANI-SSA sensor to H_2_S.

Gas-sensing properties can be explained by the response and recovery curve of PANI sensors. The whole gas-sensing response process could be divided into the following five stages: (a) the quick response stage as soon as gas molecules just reach sensitive film; (b) the slow permeate stage when gas molecules have met the film for some time; (c) the response reaches a stability or saturation stage; (d) the quick recovery stage after gas molecules get off from film; (e) recovery to the initial stage over a long time, shown in Figure 16.

Differences with regard to response time of different PANI gas sensors can be explained as follows. According to Figure 16, the response time mainly depends on the first stage, namely stage (a). Gas molecules of high concentration could quickly attach on the gas sensitive layer, which therefore shortens the time of stage (a), thus making the whole process shorter. The size of the anion pair in SSA-deposited PANI is bigger than that of HCl-deposited PANI. When the anion enters into the main chain of PANI, the structure of the chain is more stretched and larger chain spacing is formed compared with small-acid-deposited PANI. As a result, the chain-to-chain interaction of polymer is less effective, which accounts for the delocalization of charges on the molecule chain. Thereby the activity and conductivity of PANI can be enhanced and the response time decreases as a result.

Differences with regard to gas-sensing response values can be explained as follows based on Figure 14. Gas-sensing response of conductive polymer based sensors depends on the inner charge transfer, modulation of the oxidation-reduction degree, as well as the protonation effect, which result in the change of resistance rate [24]. The good sensitivity of PANI-HCl sensors to H_2_S and SO_2_ attributes to the redeposition effect of acidic gas on gas sensitive material, while the deposition degree of PANI-SSA is relatively high with an anion pair on the skeleton of PANI dispersing more uniformly. PANI-SSA produces a large delocalization degree against the redeposition effect, and thereby the sensitivity of PANI-SSA to H_2_S and SO_2_ is the lowest.

#### 3.3.2. Comparison of Stability and Mechanism Analysis

Working life time and response stability are two significant indexes to evaluate properties of sensors. From the calculation from the previous parts of this paper, the stability sequence among three type of sensors are as following: PANI-HCl/SSA (89.3%) > PANI-SSA (81.4%) > PANI-HCl (74.7%).

Mechanism analysis of the above stability can be explained as follows. For one hand, for PANI-HCl, volatilization is likely to occur after HCl deposition, thus making the previously deposited HCl move off from PANI. On the other hand, for PANI-SSA, SSA which has relatively large size and slow speed of volatilization, has both polar and non-polar groups that make PANI-SSA stable with higher decomposition temperature. Besides, due to the large size of deposited anion, interaction between molecular chains is relatively weak, which plays a role in hindering the cross-linking degradation function. Therefore, the stability of PANI-SSA is better than PANI-HCl, while the jointly-deposited PANI-HCl/SSA shows the best performance because of property enhancement of both HCl and SSA.

**Figure 17:**
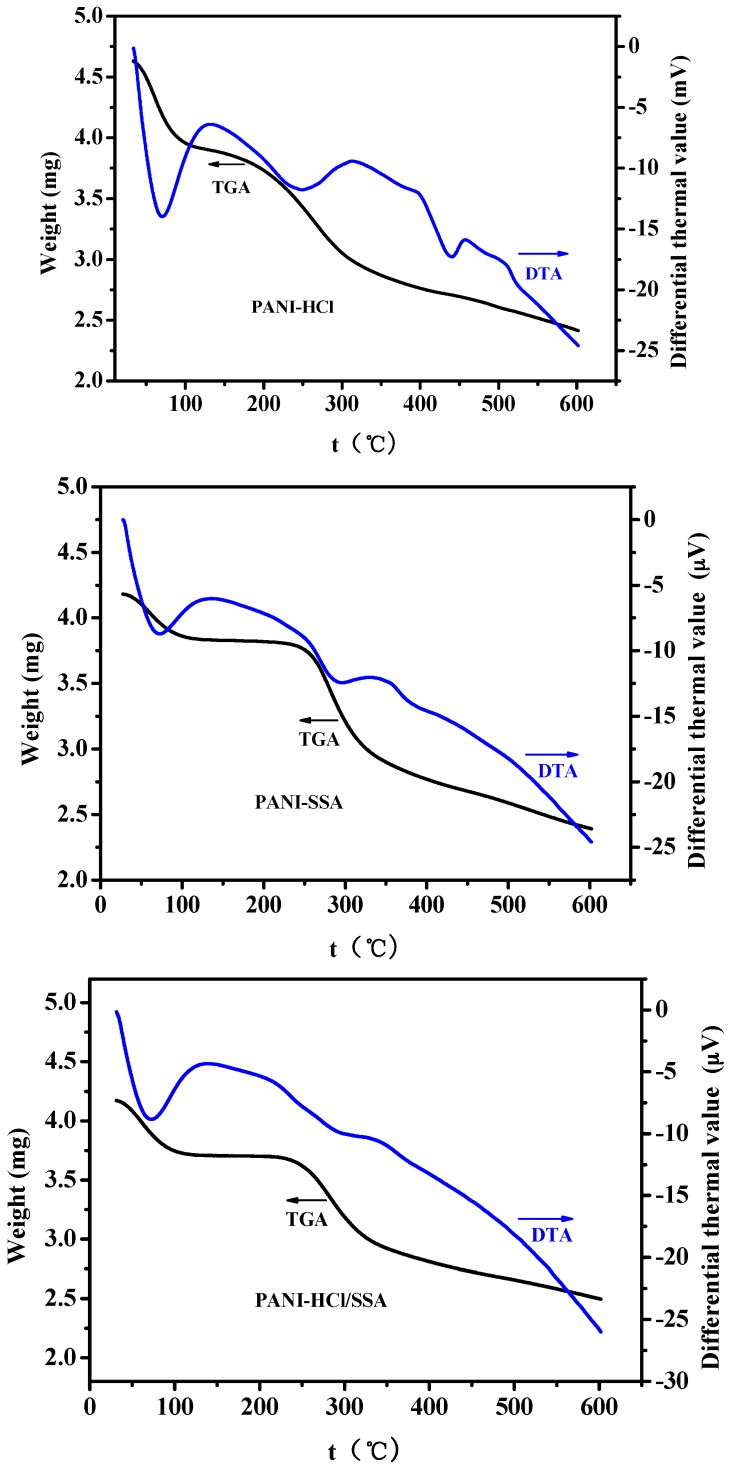
TGA-DTA curve of PANI-HCl (**top**), PANI-SSA (**middle**), and PANI-HCl/SSA (**bottom**).

Further investigation was carried out by thermogravimetric analysis (TGA) and differential thermal analysis (DTA). Figure 17 shows the curves obtained that were applied to present thermal stability of different proton acid deposited polyaniline. Three samples showed similar tendency, in which weight loss under 100 °C resulted from the evaporation of the remaining water, then the decomposition and desorption of deposited acid, followed by degradation of part of polymer chain and small amount of oligomer, finally the decomposition of the main chain of polyaniline.

Weight loss percentage of PANI-HCl, PANI-SSA, and PANI-HCl/SSA are respectively 47.9%, 42.6%, and 40.1%, which means that weight loss percentage of compound acid deposited polyaniline is the lowest under the same thermogravimetric experimental conditions. When the temperature ranges from 100 °C to 200 °C, PANI-HCl shows weight loss while the weight of the other two sensors almost remains unchanged, which is mainly because of the volatilization of HCl in PANI-HCl. When the temperature reaches around 248 °C, the PANI-HCl shows a relatively strong de-deposited endothermic peak, while the initial removal temperatures of the other two deposited protonic acid in PANI are approximately 230 °C and 240 °C. Also, it could be observed from Figure 15 that the weight loss rate of PANI-HCl/SSA is slow. So the thermostability of PANI-HCl/SSA is much better than those of PANI-HCl and PANI-SSA that were both deposited by single proton acid. Therefore, PANI-HCl/SSA sensors possess better stability performances in harsh environment.

To summarize, the properties of three types of sensors—PANI-HCl, PANI-SSA, and PANI-HCl/SSA—are concluded as follows: firstly, the sensitivity of PANI-HCl/SSA sensors to SO_2_ and H_2_S is a bit lower than that of PANI-HCl, but still shows relatively good sensitive performance; as for the response time, the response rate of PANI-HCl/SSA is larger than that of PANI-HCl and less than that of PANI-SSA; in term of working stability, PANI-HCl/SSA sensors have remarkable advantages, mainly of both heat stability and long operating stability. Thus, mixed-acid-deposited polyaniline sensors demonstrates the best overall performance although they a little inferior with respect to polyaniline of single proton acid deposition.

## 4. Conclusions

In this paper, chemical oxidative polymerization method was adopted to prepare SSA-deposited and HCl/SSA deposited PANI sensors in acid medium with aniline as the monomers and ammonium persulfate as the oxidizer. Surface characterization of the prepared gas-sensing films were carried out using FTIR, XRD, and SEM, which showed that anion pair of SSA entered into the main chain of PANI after deposition. These two newly-prepared sensors were tested for gas-sensing performances with that of HCl-deposited PANI studied in precious research work. The conclusions are as follows:

(1) The results of gas-sensing tests show that, when exposed to H_2_S and SO_2_ of various concentrations, PANI-HCl/SSA sensors show significant response compared with PANI-SSA, a little less than the response of PANI-HCl; PANI-SSA sensors respond most quickly, and PANI-HCl/SSA response is slightly slower, with PANI-HCl showing the longest response time.
(2) Thermal stability tests of TGA-DTA to these three types of sensors show that composite acids deposited PANI sensors possess better stability than single acid deposited PANI sensors, including PANI-HCl and PANI-SSA sensors. Long-term stability tests also show that PANI-HCl/SSA sensors have the best stability.
(3) The two aimed gases, that is, H_2_S and SO_2_, are generated in different stages with different production rates, which means that cross interference does not happen easily in the detection of these two gases. Considering the overall gas-sensing performances of PANI-HCl, PANI-SSA, and PANI-HCl/SSA sensors, PANI-HCl/SSA sensors are most likely to be utilized for PD detection inside GIS.

## Figures and Tables

**Table 1:** FTIR characteristic peaks of different acid-deposited PANI.

Vibration Group	PANI-HCl (cm^−1^)	PANI-SSA (cm^−1^)	PANI-HCl/SSA (cm^−1^)
N-H stretching vibration	3203	3231	3220
Quinone ring frame vibration	1559	1561	1560
Benzene skeleton vibration	1473	1489	1484
N-B-N stretching vibration	1300	1301	1296
B-NH-B	1122	1143	1120
O=S=O	-	1026	1025
Out-of-ring bending vibration of benzene ring	801	802	798
Aromatic ring bending vibration	508	505	506

**Table 2:** Gas-sensing response of different acid-deposited PANI to SO_2_ and H_2_S at low concentration.

Gas Sensors	1.0 μL/L SO_2_	0.5 μL/L H_2_S
PANI-SSA	1%~2%	about 2%
PANI-HCl/SSA	about 10%	about 20%
